# Maltodextrin-Based Cross-Linked Electrospun Mats as Sustainable Sorbents for the Removal of Atenolol from Water

**DOI:** 10.3390/polym16060752

**Published:** 2024-03-09

**Authors:** Claudio Cecone, Valentina Fiume, Pierangiola Bracco, Marco Zanetti

**Affiliations:** 1 Department of Chemistry, Nis Interdepartmental Centre, University of Turin, Via P. Giuria 7, 10125 Turin, Italypierangiola.bracco@unito.it (P.B.); 2 Instm Reference Centre, University of Turin, Via G. Quarello 15A, 10135 Turin, Italy; 3 ICxT Interdepartmental Centre, University of Turin, Via Lungo Dora Siena 100, 10153 Turin, Italy

**Keywords:** electrospinning, dextrins, biobased sorbents, emerging pollutants, decontamination

## Abstract

Maltodextrins are products of starch hydrolysis that can be processed into dry fibres through electrospinning and subsequently cured via mild thermal treatment to obtain nonwoven cross-linked polysaccharide-based mats. The sustainability of the process and the bioderived nature make this class of materials suitable candidates to be studied as renewable sorbents for the removal of contaminants from water. In this work, electrospinning of water solutions containing 50% wt. of commercial maltodextrin (Glucidex 2^®^) and 16.6% wt. of citric acid was carried out at 1.2 mL/h flow and 30 kV applied voltage, followed by thermal curing at 180 °C of the dry fibres produced to obtain cross-linked mats. Well-defined fibres with a mean diameter of 1.64 ± 0.35 µm were successfully obtained and characterised by scanning electron microscopy, thermogravimetric analysis, and attenuated total reflectance Fourier transform infrared spectroscopy. Afterwards, a series of sorption tests were conducted to evaluate the effectiveness of the mats in removing atenolol from water. The results of the batch tests followed by HPLC-UV/Vis showed high sorption rates, with over 90% of the atenolol removed, and a maximum removal capacity of 7 mg/g. Furthermore, continuous fixed-bed sorption tests proved the positive interaction between the polymers and atenolol.

## 1. Introduction

The use of polysaccharides as sustainable alternatives to fossil-based plastics has gained increasing scientific and industrial interest, aiming to move toward a circular economy [1,2]. In this context, starch and cellulose have been applied in the packaging industry and for bio-composite production [3,4,5,6,7]. They have also been exploited in various fields such as food, pharmaceuticals, environmental science, and medical science [8,9,10,11,12,13,14,15,16]. Produced by enzymatic conversion from starch, maltodextrins are D-glucose-based water-soluble polymers, used mainly as pharmaceutical and food additives [17]. The dextrose equivalent (DE) is a measure of the degree of polymerisation of each maltodextrin, which is determined by its reducing equivalent against the same glucose mass. The DE for maltodextrins is usually lower than 20 [18]. Maltodextrins have been utilised as affordable bioderived building blocks to create cross-linked polymers [19,20,21,22,23,24,25,26]. Furthermore, maltodextrins have been successfully processed via electrospinning to obtain polysaccharide-based fibres [27].

Electrospinning is a cost-effective, facile, and flexible processing technique that allows the production of fibres from electrically charged jets of polymer solutions [28]. The strength of this technique is associated with the high surface-to-volume ratio of the mats produced, which are characterised by fibres with diameters ranging from micrometres to nanoscale [29,30,31]. However, the use of toxic or flammable organic solvents is often needed to dissolve the polymer, which limits industrial production due to environmental and safety regulations [32,33,34,35,36,37]. For this reason, the reduction or substitution of hazardous substances is an active research field, in agreement with the Green Chemistry principles [38,39]. In this framework, the electrospinning of maltodextrins using water and ethanol/water mixtures as the solvent media was reported by Stijnman et al. [27] and Vargas-Campos et al. [40], respectively. Maillard reactions have been exploited to cross-link electrospun protein-containing maltodextrin fibres, as demonstrated by Kutzli et al. and Gibis et al. [41,42,43,44]. Similarly, Ruggeri et al. cross-linked maltodextrin amino acid electrospun scaffolds for skin tissue engineering [45]. Environmental studies related to the use of polysaccharide electrospun fibres were carried out by Huang et al. [46] and Nthunya et al. [47], who exploited chitosan-based fibres to remove metal ions and phenols, respectively. Recently, Mantripragada et al. [48] reported the use of soy protein-coated cellulose-based electrospun membranes for the remediation of emerging contaminants from water. 

Emerging contaminants are composed of different classes of compounds, such as active personal care products, pharmaceutical ingredients, pesticides, and plasticisers. There is concern regarding their presence in the environment. This is mainly due to experimental data showing their possible aptitude to cause negative outcomes on flora, fauna, and human health. Additionally, these substances are difficult to degrade and are resistant to common water treatment processes [49,50,51,52,53]. Pharmaceuticals such as β-blockers are found in the environment at low but meaningful levels. While not highly persistent, many are considered “pseudo-persistent” due to continuous discharge into the environment from various sources [54,55]. Atenolol is one of the most widely used β-blockers, mainly in the treatment of cardiovascular conditions including abnormal heart rhythms, high blood pressure, and angina pectoris [56,57]. High prescription rates and incomplete metabolism can lead to the presence of atenolol in municipal wastewater effluents up to the µg/L scale, causing the potential for its deposition into aquatic ecosystems and exposure consequences for aquatic wildlife [58,59]. In this work, nonwoven mats were first obtained by the electrospinning technique and then cross-linked via a mild thermal treatment. Subsequently, the cured fibres were tested for their potential to remove atenolol, an emerging pollutant, from water.

## 2. Experimental

### 2.1. Materials

Maltodextrins with a DE value of 2 (Glucidex 2^®^, GLU2) were provided by Roquette Freres (Lestrem, France), while citric acid (99%) (CIT) and atenolol (AT) were purchased from Sigma-Aldrich (Darmstadt, Germany). GLU2 was dried in an oven at 75 °C, for 24 h, to reach the complete evaporation of the water adsorbed, i.e., constant weight of the material.

### 2.2. Sample Preparation

In a typical procedure, 0.5 g of CIT was dissolved in 3.0 mL of distilled water under stirring at 70 °C. Once the solubilization was complete, 3.0 g of GLU2 was added to the solution. After approximately 30 min, a homogeneous solution was obtained. The sample was then degassed in a heated ultrasound bath for around 15 min at 70 °C and allowed to cool to room temperature. The final concentration of maltodextrin in water was 50% wt., and the CIT/GLU weight ratio was 1:6.

### 2.3. Processing and Curing

An electrospinning device made from a 3 mL syringe, a power supply, and a volumetric pump was used to process the GLU2 solutions. A nozzle of gauge 18, a working distance of 15 cm, a field strength of 30 kV, and a flow rate of 1.2 mL/h were set. The deposition was performed at a relative humidity of 30% to 45% and room temperature. An aluminium cylinder-equipped rotary system (Linari NanoTech Easy Drum from Pisa, Italy) was used as the collector, with a rotation speed of 75 rpm. The mats were cured at 180 °C in an oven for 30 min.

### 2.4. Solubility Test

Polymer mats weighing 50 mg were transferred into a 5 mL plastic container. Then, 5 mL of deionised water was added and the samples were left to rest at room temperature for 15 h. After that, the solution was removed from the mat via centrifugation, and the mat was then dried in an oven at 60 °C until it reached a constant weight. Each test was performed in triplicate. The soluble fraction was obtained using the following formula,

(1) $$Soluble\;fraction\left(\%\right)=\left(\frac{{SMP}_{before}-{SMP}_{after}}{{SMP}_{before}}\right)*100$$

where *SMP_before_* represents the weight of the mat (mg) before the test and *SMP_after_* the weight of the mat (mg) after the test.

### 2.5. Thermogravimetric Analysis (TGA)

TGA was carried out under a nitrogen flow of 100 mL/min with a Q500 TGA, TA Instruments (New Castle, DE, USA), employing 10 mg of sample and a heating rate of 10 °C/min from 50 °C to 700 °C.

### 2.6. Attenuated Total Reflectance Fourier Transform Infrared (FTIR-ATR) Spectroscopy

The spectra were collected with a PerkinElmer (Waltham, MA, USA) spectrometer at room temperature within the wavenumber range of 650–4000 cm^−1^, with a resolution of 4 cm^−1^ and 8 scans per spectra.

### 2.7. Scanning Electron Microscopy (SEM)

A Tescan VEGA 3 SEM (Brno, Czech Republic) was used. The samples, previously coated with 12 nm of gold, were imaged with secondary electrons at an accelerating voltage of 5–6 kV. ImageJ (version 1.51j8) was used to manually evaluate the diameter distribution. Reliable statistics were generated by performing 100 measurements for each SEM image without considering those fibres lying outside the focal plane.

### 2.8. Atenolol Sorption Tests and HPLC-UV/Vis Detection

Batch sorption tests were conducted using 25 mL of 1 mg/mL and 10 mg/L AT water solutions at room temperature and pH values of 6, 7, and 8. The quantity of polymer used for the sorption tests was 1 mg/mL, 2 mg/mL, or 4 mg/mL, concerning the AT solutions, while an orbital shaker was used to continuously stir the vials at room temperature. A standard curve was created to measure the concentration of AT in the range between 0.1 mg/L and 1 mg/L for the experiments conducted at 1 mg/L, and another standard curve was constructed to measure the concentration of AT in the range between 1 mg/L and 10 mg/L for the experiments conducted at 10 mg/L. The concentration of AT was measured at fixed intervals using HPLC separation and UV/Vis detection. The instrument was a Dionex (Sunnyvale, CA, USA), composed of a pump P680 and a detector UVD170U. Separation was achieved using a Phenomenex (Torrance, CA, USA) Kinetex^®^ C18 (150 × 4.6 mm, 5μm) column, and the mobile phase consisted of 0.1% orthophosphoric acid buffer and acetonitrile in a ratio of 90:10 *v*/*v*. The mobile phase was filtered using a 0.45 µm nylon filter and degassed before use. The run time for the experiment was 5 min with a retention time of 3.5 min for AT and a flow rate of 1 mL/min. The quantification of AT was performed at 230 nm, and the detection limit was 0.1 mg/L. The effectiveness of spun mats in removing AT from water solutions was measured every 15, 30, 60, 120, and 180 min and expressed as the percentage of sorption (*Sor_(%)_*):

(2) $${Sor}_{\left(\%\right)}=\left(1-\frac{{Conc\;t}_{x}}{{Conc\;t}_{0}}\right)\times 100$$

In parallel, the sorption capacity was determined, which is the number of milligrams of AT removed per gram of sorbent (*Sor_(mg/g)_*):

(3) $${Sor}_{\left(mg/g\right)}=\left({Conc\;t}_{0}-{Conc\;t}_{x}\right)\times \frac{V}{m}$$

where *Conc t*_0_ (mg/L) represents the initial concentration of AT, *Conc t_x_* (mg/L) is the concentration of AT after each time interval, *V* (L) is the volume of the AT solution, and *m* (g) is the mass of sorbent used. 

Continuous fixed-bed sorption experiments were conducted using a custom-built apparatus comprising a six-step plastic column filled with mat portions with a diameter of 16 mm and a total weight of 45 mg. A drip funnel containing a 2.5 mg/mL AT solution was joined to the upper region of the column, and the flow of permeation, gravity-induced, was set to 0.5 mL/min. Detection via HPLC-UV/Vis was performed on every 5 mL of solution that passed through the column, following the previously described method. Three trials were performed for each sorption experiment.

## 3. Results and Discussion

### 3.1. Deposition and Characterisation of the Mats

**Figure 1:**
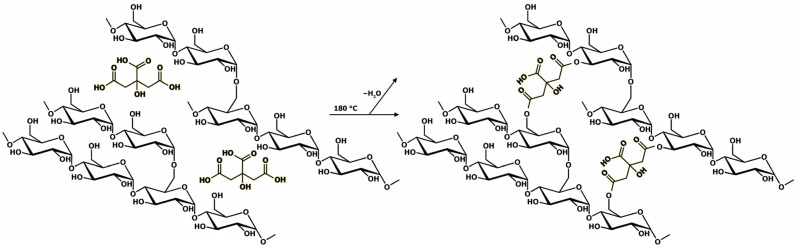
The cross-linking reaction between GLU2 and CIT.

As demonstrated in a recent study by our group [60], GLU2 can be easily electrospun into dry and homogeneous fibres from water solutions containing 50 wt%. maltodextrin. However, the intrinsic water solubility of GLU2 hinders the possibility of employing such spun fibres for applications in the presence of water or moisture. For this reason, it was demonstrated that the incorporation of citric acid in the GLU2 water solution does not alter the resulting fibrous morphology and allows the spun mat to be thermally cured at 180 °C, after the deposition process, obtaining a cross-linked material. The curing reaction was shown to occur between the hydroxyl groups belonging to the maltodextrin chains and the molecules of CIT (Figure 1), generating ester bridges and the release of water as a reaction byproduct, as observed via TGA and FTIR-ATR. With this in mind, the first part of this work focused on the production and curing of GLU2-based CIT-linked fibrous mats.

**Figure 2:**
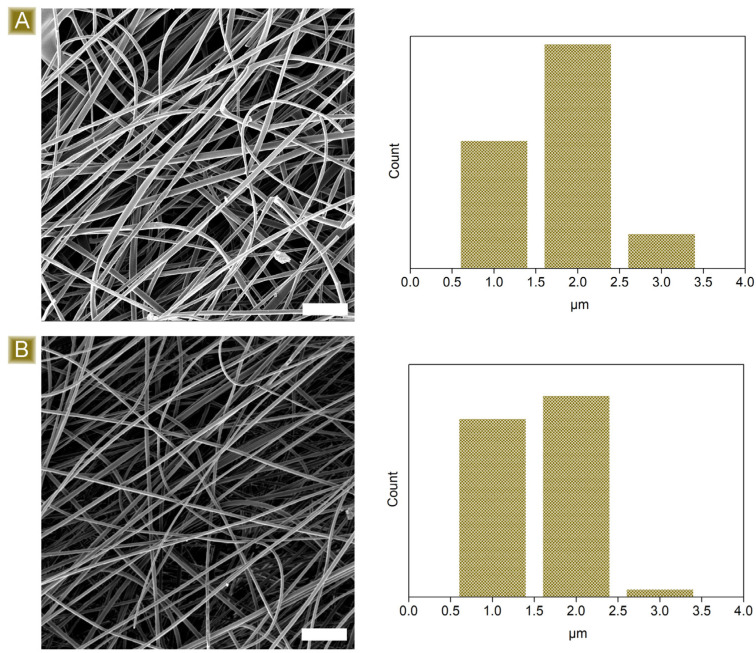
Morphological characterisation of the spun fibres (**A**) before and (**B**) after thermal curing. Scale bars: 20 µm.

**Figure 3:**
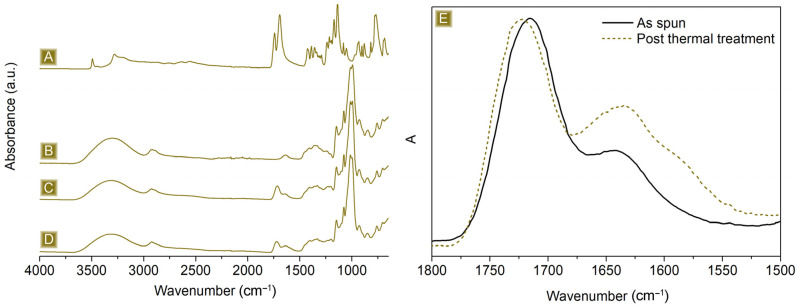
FTIR-ATR of (**A**) CIT, (**B**) GLU2, (**C**) as-spun mat, and (**D**) spun mat after thermal treatment. (**E**) Details of the carbonyl signal, range: 1800–1500 cm^−1^.

As reported in Figure 2, the deposition of nonwoven mats, characterised by well-defined fibres, displaying a mean diameter of 1.82 ± 0.51 µm, was obtained. Typical IR signals, belonging to both dextrins and the linker, were detected in the FTIR-ATR analyses presented in Figure 3A–C. A broad band in the wavelength region 3000–3500 cm^−1^ and a signal at 1645 cm^−1^ were associated with the stretching modes and the bending signal of OH, respectively. In the region 1080–1000 cm^−1^, vibrations belonging to C–O–C or C–O groups were visible. Typical C–H stretching modes were observed at 2921 cm^−1^ and 2867 cm^−1^, while the carbonyl peak centred at 1716 cm^−1^ and the shoulder at 1178 cm^−1^ confirmed the presence of CIT molecules together with GLU2.

**Figure 4:**
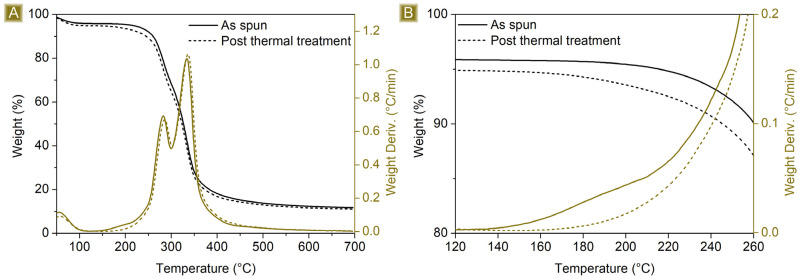
(**A**) TGA (black) and related DTGA (brown) of as-spun mat (solid) and (dot) spun mat post thermal treatment. (**B**) Details of the temperature interval.

The thermogravimetric profile in Figure 4 depicts the weight loss behaviour of the spun mat material as a function of the temperature. The first weight loss event occurs at around 125 °C, which is due to the evaporation of the adsorbed water, approximately 7% wt. of the total weight. The spun mat is thermally stable up to 200 °C, with a T_onset_ of 260 °C. Between 200 °C and 400 °C, the sample undergoes a two-step weight loss process, which is associated with its degradation, leading to the formation of a stable carbon residue of around 16 wt%. of the starting weight at 700 °C. The derivative curve shows a weight loss phenomenon occurring at around 180 °C. This is because water is eliminated as a product of the esterification reactions between citric acid and maltodextrin molecules. This reaction is essential for the cross-linking step needed to provide the material water stability. As a confirmation, this phenomenon is no longer present after thermal curing carried out at 180 °C, and the related FTIR-ATR spectrum, reported in Figure 3D,E, shows a peak shift correlated with the carbonyl stretching, from 1716 cm^−1^ to 1726 cm^−1^, as a result of the different chemical surroundings.

Further confirmation of the successful cross-linking of the spun fibres via thermal treatment was obtained from the solubility tests performed in water at room temperature. After 15 h of testing, 83.5 ± 2.4% of the initial material was recovered, assessing its relative stability. Furthermore, as reported in Figure 2B, the thermal cross-linking treatment carried out at 180 °C does not alter fibrous morphology. The fibres still appear well-defined and display a mean diameter of 1.64 ± 0.35 µm.

### 3.2. AT Batch Sorption Test

**Figure 5:**
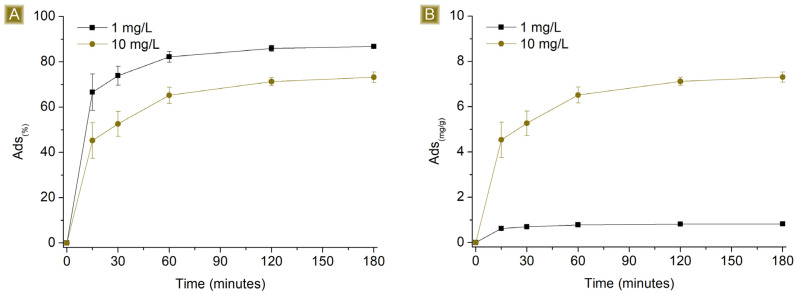
Profiles of (**A**) *Sor_(%)_* and (**B**) *Sor_(mg/g)_* of 25 mg of sorbent in 25 mL of AT solution by increasing the concentration of AT vs. time.

Time-dependent sorption of AT was observed in Figure 5, which rapidly increased over the first hour, reaching a plateau beyond 120 min of contact time between the mat and the AT solution. The highest *Sor_(%)_*, from 1 mg/L AT solutions, resulted in 86.8 ± 0.2%, with *Sor_(mg/g_*_)_ corresponding to 0.83 ± 0.01 mg/g. In the case of 10 mg/L AT solutions, the highest *Sor_(%)_* was 73.2 ± 2.3%, with *Sor_(mg/g)_* corresponding to 7.31 ± 0.23 mg/g. The ability of the mat to remove AT from water may be related to the presence of residual free carboxylic functions belonging to CIT on the spun fibres, as reported in Figure 1 and confirmed via FTIR-ATR, which can generate electrostatic interactions with AT molecules. Additionally, the kinetic profile can be related to the hydrophilic nature of the spun material. When the mats come in contact with aqueous media, the solution penetrates the inner parts of the fibres, where electrostatic interactions can form as a function of the diffusion of the solution during that time. As a consequence, the removal of AT via electrostatic interaction is a phenomenon not limited to the surface of the sorbent.

The *Sor_(%)_* was higher in the case of 1 mg/L AT solutions, while the *Sor_(mg/g)_* was higher in the case of 10 mg/L AT solutions. If we consider a given number of active sites available on a determined and constant mass of the sorbent, they will display higher *Sor_(%)_* values for a diluted solution, namely those cases in which the saturation of the sorbent is not achieved. In other words, higher *Sor_(%)_* values are reached when the number of active sites of the sorbent is higher than the moles of the target molecule, in this study pertaining to the solution with an AT concentration of 1 mg/L. In contrast, the presence of free active sites on the sorbent, after the sorption step, will result in lower *Sor_(mg/g)_* compared to those cases in which all the sites are exploited. On the other hand, with more concentrated solutions, or solutions where the moles of the target molecule are higher than the number of active sites displayed by the sorbent, saturation will occur, resulting in lower *Sor_(%)_* but higher *Sor_(mg/g)_*, as confirmed by the results observed from AT solutions of 10 mg/L.

**Figure 6:**
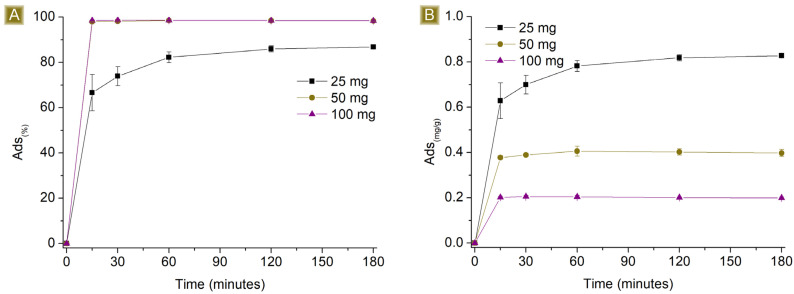
Sorption profiles (**A**) *Sor_(%)_* and (**B**) *Sor_(mg/g)_* of increasing amounts of fibres in 25 mL of 1 mg/L AT solution vs. time.

**Figure 7:**
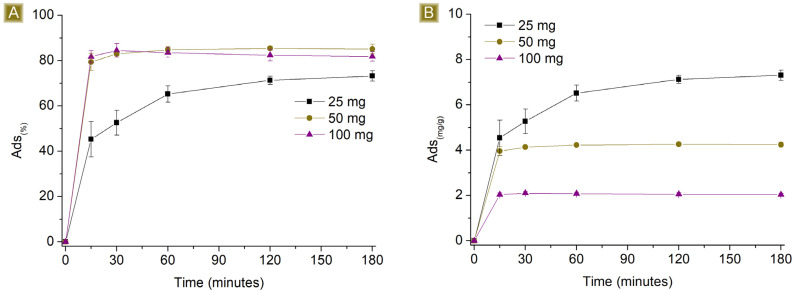
Sorption profiles (**A**) *Sor_(%)_* and (**B**) *Sor_(mg/g)_* of increasing amounts of fibres in 25 mL of 10 mg/L AT solution vs. time.

Figure 6 and Figure 7 report the performance of the spun fibres on the removal of AT, detected by changing the amount of sorbent in 25 mL of solution, at AT concentrations of 1 mg/L and 10 mg/L respectively, at pH 7.

Higher *Sor_(%)_* but lower *Sor_(mg/g)_* values were observed by increasing the amount of sorbent. The observed results can be attributed to two factors, namely the electrostatic nature of the sorption and the proportion of the number of active sites present on the polymer and the moles of AT available in the solution. Assuming that all the sites display similar activity, their activity towards AT will continue until they become saturated. If the number of active functionalities exceeds the amount of AT, complete removal will be detected. However, when an excess of active sites are present, despite complete removal, a decrease in the *Sor_(mg/g)_* will be obtained, proportional to the excess of active sites. When there are more moles of AT than active sites, residual AT will still be detectable in the solution. Reasonably, if electrostatic interactions are the only phenomena taking place, the best performance in terms of *Sor_(mg/g)_* will be observed upon reaching the saturation of the active sites. After conducting tests with different amounts of sorbent, we found that the *Sor_(%)_*_,_ values at a contact time of 180 min were total for 100 mg and 50 mg and equal to 86.8 ± 0.2% for 25 mg of sorbent. Additionally, with higher amounts of fibres, the sorption plateau was reached more quickly as a consequence of the increasing number of easily accessible active sites displayed by the sorbent. With 100 mg and 50 mg, a few minutes were enough to reach the plateau, reasonably related to a sufficient number of active sites exposed on the surface of the mat to reach a total removal of AT. In contrast, using 25 mg of fibres results in a slower removal process, possibly due to the rate of solution permeation within the sorbent matrix and the subsequent interaction of AT with the sites accessible upon saturation of the external layers. The *Sor_(mg/g)_* was 0.83 ± 0.01 mg/g when 25 mg of fibres was used.

**Figure 8:**
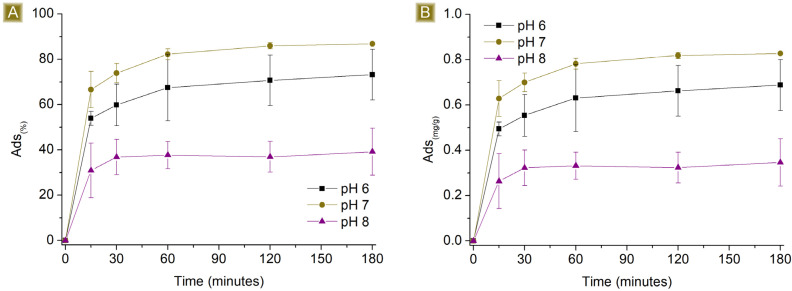
Sorption profiles (**A**) *Sor_(%)_* and (**B**) *Sor_(mg/g)_* of 25 mg of sorbent in 25 mL 1 mg/L AT solution by changing the pH of the solution vs. time.

**Figure 9:**
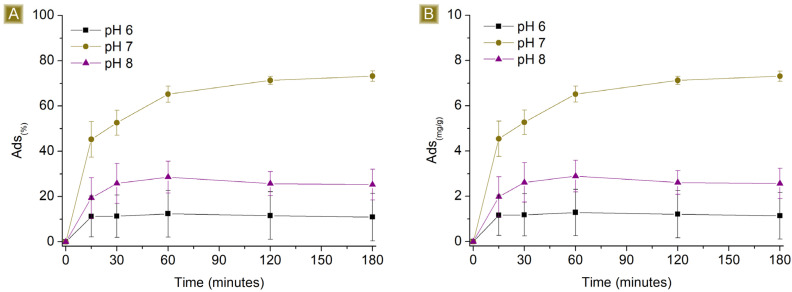
Sorption profiles (**A**) *Sor_(%)_* and (**B**) *Sor_(mg/g)_* of 25 mg of sorbent in 25 mL 10 mg/L AT solution by changing the pH of the solution vs. time.

The sorption performances of the spun mat toward AT were subsequently studied at different pH values (Figure 8 and Figure 9). The pH of the AT solution was adjusted from 6 to 8 by slowly adding either HCl 0.1M or NaOH 0.1M. The amount of sorbent used was kept at a constant 25 mg, the final volume at 25 mL, and the AT concentration at either 1 mg/L or 10 mg/L. The percentage of sorption was found to be affected at both acidic and basic pH levels, indicating that the sorption process is dependent on the pH values used. At acidic pH values, the performance decreased from 86.8 ± 0.2% to approximately 73% for 1 mg/L AT solutions, and from 73.2 ± 2.3% to roughly 10% for 10 mg/L AT solutions. The pKa of AT (9.6) shows the presence of AT in its protonated form at this pH value, a condition that favours interaction with the carboxyl functions of the sorbent. However, lower acidic pH conditions also involve the presence of higher quantities of protons in the solution. The latter has higher mobility than AT and can compete for interaction with active sites of the polymer, causing a drop in AT removal performance. At basic pH values, the performance dropped from 86.8 ± 0.2% to approximately 39% for 1 mg/L AT solutions and from 73.2 ± 2.3% to roughly 25% for 10 mg/L AT solutions. In this case, the pH favours the presence of deprotonated carboxyl groups, which belong to the sorbent, associated with stronger electrostatic interaction with cationic molecules. However, the protonation equilibrium of AT results in decreased shift toward the protonated form, resulting again, as observed in acidic pH values, in a decrease in sorption performance.

**Figure 10:**
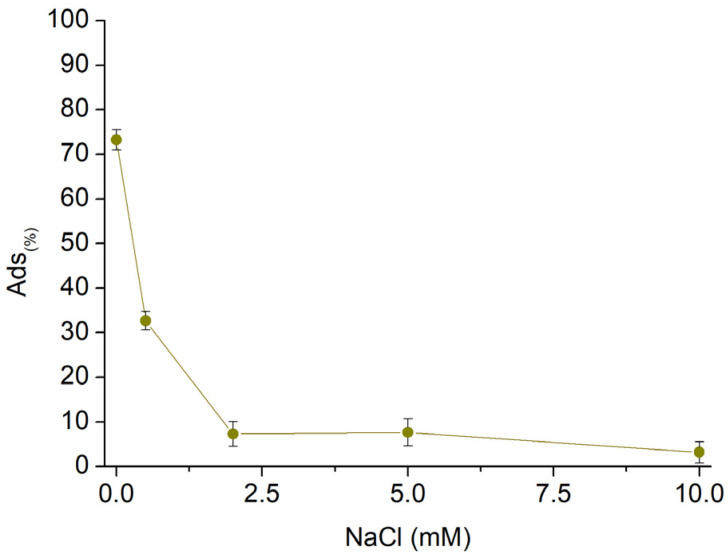
Sorption profile vs. increasing NaCl concentration.

Subsequently, to assess the AT removal capability in nonideal conditions, sorption tests were conducted on AT solutions with varying concentrations of NaCl (Figure 10). The concentration of NaCl dissolved in the AT solution was increased from 0.5 mM to 10 mM while keeping the concentration of AT at 10 mg/L, the final volume of 25 mL, and the quantity of fibres at 25 mg. The presence of salts in the solution affected the sorption performances, proving how the electrostatic interactions represent the main phenomena characterising the sorption mechanisms. The effect of NaCl resulted in a decrease in sorption performance proportional to the salt concentration. Until a concentration of NaCl equal to 0.5 mM, which corresponds to 0.3 mg/L, *Sor_(%)_* values higher than 30% were observed. Furthermore, the performance decreased below 10% for NaCl 2.0 mM (1.2 mg/L). The increasing concentration of sodium cations in solution, related to the increasing amount of NaCl, caused the latter to compete for the active sites of the sorbent, with a resulting drop in the sorbed AT quantities.

### 3.3. AT Fixed Bed Sorption Test

**Figure 11:**
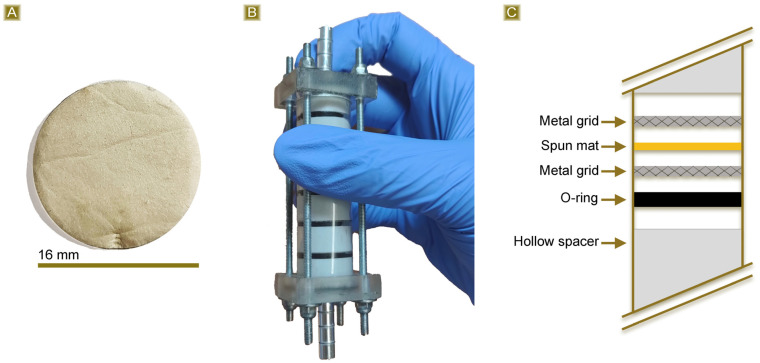
(**A**) Circular specimens obtained from the cured spun mat, (**B**) multistep column used for the fixed bed sorption test, and (**C**) scheme reporting the section of the column.

**Figure 12:**
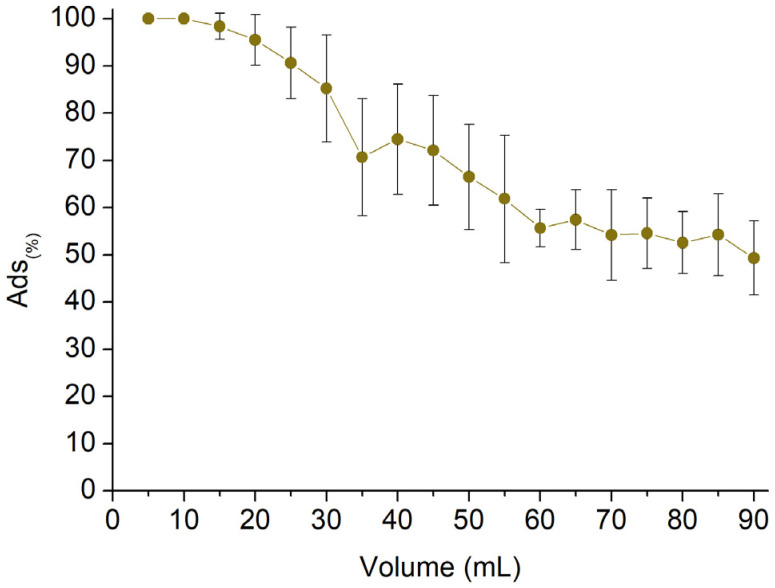
Column sorption evaluation of 2.5 mg/L AT solution employing 45 mg of sorbent.

The last part of the work was carried out to evaluate the potential applicability of the reported fibrous mats in continuous sorption scenarios. Circular samples with a diameter of 16 mm (Figure 11A) were obtained from the thermally treated spun mat and subsequently placed in a multistep column (Figure 11B). The column is composed of six steps, each consisting of one disc of mat confined between two metal grids and an O-ring to ensure flow passage through the membrane (Figure 11C). Each step is separated from the others with a hollow spacer, and the total amount of membrane is 45 mg. The test was carried out using a 2.5 mg/L AT solution. The AT concentration was monitored as the latter permeated through the mats at a flow rate of 0.5 mL/min, with measurements taken every 5 mL of solution withdrawn. Figure 12 shows the *Sor_(%)_* reported in response to the permeated volume. The results indicate that the sorption percentage was higher than 90% during the first 25 mL of withdrawn solution and remained over 60% up to 55 mL of recovered solution. However, the sorbent removal capacity subsequently decreased to approximately 50% between 60 and 90 mL of permeate solution. The test was stopped at this point. Taking into account the volume, the amount of sorbent, and the concentration of AT, a *Sor_(mg/g)_* equal to 6.30 mg/g was obtained. This value shows good agreement with the highest *Sor_(mg/g)_* value observed from the batch tests, which was equal to 7.31 mg/g.

## 4. Conclusions

Glucidex 2^®^ (GLU2), a commercial maltodextrin, was solubilised in distilled water together with citric acid (CIT) and successfully electrospun into dry nonwoven mats. The presence of citric acid allowed the produced fibres to be cross-linked via a mild thermal treatment, and the mats were subsequently used as suitable sorbents for the removal of atenolol (AT) from aqueous solutions. The mats were morphologically characterised via SEM, showing well-defined fibres with a mean diameter of 1.64 ± 0.35 µm. Furthermore, the mats were found to be thermally stable up to 250 °C, according to the T_onset_ calculated from the related TGA. In addition, the occurrence of cross-linking reactions was confirmed through both water solubility tests and FTIR-ATR measurements. From the first AT sorption test, the presence of free carboxyl functions belonging to CIT, involved in the formation of bridges between GLU2 chains, was related to the generation of electrostatic interactions between fibres and AT, thus pivotal in affecting the removal of the latter. Based on the experiments conducted, it was found that the use of 2.0 mg of sorbent per mL of 1 mg/L AT solution resulted in a removal rate of more than 90% and a sorption capacity of 0.40 mg/g. On the other hand, when dealing with a 10 mg/mL AT solution, using only 1.0 mg of sorbent per mL of solution can achieve a removal efficiency of 73% and a sorption capacity of 7.31 mg/g. During the sorption tests, the optimal pH value was found to be 7 considering both AT and carboxyl groups’ protonation equilibria and the competition with protons. The presence of salts in the solution hindered the sorption of AT, indicating that these sorbents are better suited for decontaminating low-salinity waters. To demonstrate the efficacy of these sorbents in continuous water treatment applications, fixed-bed sorption tests were also performed. Circular specimens obtained from the cross-linked spun mat were placed in a multistep column and a 2.5 mg/L AT solution was allowed to permeate at 0.5 mL/min. During the test, 45 mg of spun mat showed a removal rate of over 90% for the first 20 mL permeated. However, the rate gradually dropped to 50% as the test progressed until finally 90 mL of solution permeated. The sorption capacity was calculated to be 6.30 mg/g.

## Data Availability

Data are contained within the article.
